# Genomic Sequencing of *Orientia tsutsugamushi* Strain Karp, an Assembly Comparable to the Genome Size of the Strain Ikeda

**DOI:** 10.1128/genomeA.00702-16

**Published:** 2016-08-18

**Authors:** Hsiao-Mei Liao, Chien-Chung Chao, Haiyan Lei, Bingjie Li, Shien Tsai, Guo-Chiuan Hung, Wei-Mei Ching, Shyh-Ching Lo

**Affiliations:** a Division of Cellular and Gene Therapies, Office of Cellular, Tissue and Gene Therapies, Tissue Microbiology Laboratory, Center for Biologics Evaluation and Research, U.S. Food and Drug Administration, Silver Spring, Maryland, USA; b Naval Medical Research Center, Silver Spring, Maryland, USA; c Uniformed Services University of the Health Sciences, Bethesda, Maryland, USA

## Abstract

*Orientia tsutsugamushi*, an intracellular bacterium, belongs to the family *Rickettsiaceae*. This study presents the draft genome sequence of strain Karp, with 2.0 Mb as the size of the completed genome. This nearly finished draft genome sequence was annotated with the RAST server and the contents compared to those of the other strains.

## GENOME ANNOUNCEMENT

*Orientia tsutsugamushi* is an obligate intracellular organism causing scrub typhus. The disease in endemic triangle influences more than one billion people in the world (1). The genome size among the strains is varied; only two completed genomes, those of strain Boryong and Ikeda, have been published (2, 3). The genome of *Orientia tsutsugamushi* contains near 40% identical repeats, which troubles the sequence assembly. In this study, we combined a cloned-based method and high-throughput sequencing to assemble the draft genome of strain Karp, which was originally isolated from Papua New Guinea (4, 5).

Clone-based whole-genome shotgun sequencing generated the reads by sequencing bacterial artificial chromosome (BAC) clones with the Sanger method. The assembled reads formed 116 contigs before high-throughput whole-genome sequencing (HTGS) was available; the contig length ranged from 1,153 to 151,627 bp. The raw reads of HTGS were used to join the contigs from the Sanger read assembly, and the original 116 contigs were reduced to 99. HTGS was conducted using the Illumina MiSeq platform with the 2 × 250 bp paired-end mode. The trimmed raw reads were *de novo* assembled into an original 1,011 contigs using CLC Genomics Workbench 9.0, with an average coverage of 73×. The contigs from HTGS were first mapped to three references: (i) the contig set from the Sanger assembly, (ii) strain Boryong (accession no. NC_009488.1), and (iii) strain Ikeda (accession no. NC_010793.1), and subjected to a BLAST search against the nt/nr database to eliminate contigs from background DNA contaminations. The raw reads retrieved from filtered contigs were *de novo* assembled and mapped again to the three references, plus a published Karp draft genome (GenBank accession number NZ_LANM00000000.1) for the secondary background cleaning. All retained HTGS contigs of strain Karp can be mapped to at least one of the references, and five contigs from HTGS (combined length, 11,304 bp) were added to the original 99 Sanger contigs (2,011,605 bp) to form the draft genome of 2,022,909 bp (30.41% G+C content). The 104 (99 + 5) contigs of Karp were aligned to the Ikeda complete genome using the CONTIGuator software (6).

The Karp genome was annotated using RAST server version 2.0 (7), with 2,089 coding sequences (CDSs), 1,090 transcribed from the positive strand, and 999 transcribed from the negative strand; 2,052 of these were categorized into 185 subsystems, and 37 were RNAs. A SEED Viewer sequence comparison (8) showed that 1,604 genes of strain Boryong and 1,951 genes of strain Ikeda could be found in the Karp draft genome. The HTGS was also conducted to sequence the other two *O. tsutsugamushi* strains, AFSC4 and AFSC7, without the information about the repetitive sequences assembled from cloned-based sequencing. The SEED Viewer functional comparison (8) revealed that two genes presenting in both AFSC4 and AFSC7 were absent in Karp, even though it possesses more CDSs in its genome (data not shown). Previous studies showed that strain Karp was sensitive to antibiotics, whereas AFSC4 was insensitive (9), and AFSC7 had similar internal observations. A completed draft genome of AFSC4 and AFSC7 for delicate comparison with the Karp genome may provide the possible targets for investigation of microbial drug resistance mechanism(s).

### Accession number(s).

The first version of draft genome sequences of *Orientia tsutsugamushi* strain Karp was deposited in GenBank under accession no. LYMA00000000.

## References

[1] Kelly DJ, Fuerst PA, Ching WM, Richards AL 2009 Scrub typhus: the geographic distribution of phenotypic and genotypic variants of *Orientia tsutsugamushi*. Clin Infect Dis 48(Suppl 3):S203–S230. doi:10.1086/596576. 19220144

[2] Cho NH, Kim HR, Lee JH, Kim SY, Kim J, Cha S, Kim SY, Darby AC, Fuxelius HH, Yin J, Kim JH, Kim J, Lee SJ, Koh YS, Jang WJ, Park KH, Andersson SG, Choi MS, Kim IS 2007 The *Orientia tsutsugamushi* genome reveals massive proliferation of conjugative type IV secretion system and host-cell interaction genes. Proc Natl Acad Sci USA 104:7981–7986. doi:10.1073/pnas.0611553104. 17483455 PMC1876558

[3] Nakayama K, Yamashita A, Kurokawa K, Morimoto T, Ogawa M, Fukuhara M, Urakami H, Ohnishi M, Uchiyama I, Ogura Y, Ooka T, Oshima K, Tamura A, Hattori M, Hayashi T 2008 The whole-genome sequencing of the obligate intracellular bacterium *Orientia tsutsugamushi* revealed massive gene amplification during reductive genome evolution. DNA Res 15:185–199. doi:10.1093/dnares/dsn011. 18508905 PMC2575882

[4] Derrick E, Brown HE 1949 Isolation of the Karp strain of *Rickettsia tsutsugamushi*. Lancet 254:150–151. doi:10.1016/S0140-6736(49)90996-4. 18146951

[5] Odorico DM, Graves SR, Currie B, Catmull J, Nack Z, Ellis S, Wang L, Miller DJ 1998 New *Orientia tsutsugamushi* strain from scrub typhus in Australia. Emerg Infect Dis 4:641–644. doi:10.3201/eid0404.980416. 9866742 PMC2640248

[6] Galardini M, Biondi EG, Bazzicalupo M, Mengoni A 2011 CONTIGuator: a bacterial genomes finishing tool for structural insights on draft genomes. Source Code Biol Med 6:11. doi:10.1186/1751-0473-6-11. 21693004 PMC3133546

[7] Overbeek R, Olson R, Pusch GD, Olsen GJ, Davis JJ, Disz T, Edwards RA, Gerdes S, Parrello B, Shukla M, Vonstein V, Wattam AR, Xia F, Stevens R 2014 The SEED and the rapid annotation of microbial genomes using subsystems technology (RAST). Nucleic Acids Res 42:D206–D214. doi:10.1093/nar/gkt1226. 24293654 PMC3965101

[8] Overbeek R, Begley T, Butler RM, Choudhuri JV, Chuang HY, Cohoon M, de Crécy-Lagard V, Diaz N, Disz T, Edwards R, Fonstein M, Frank ED, Gerdes S, Glass EM, Goesmann A, Hanson A, Iwata-Reuyl D, Jensen R, Jamshidi N, Krause L, Kubal M, Larsen N, Linke B, McHardy AC, Meyer F, Neuweger H, Olsen G, Olson R, Osterman A, Portnoy V, Pusch GD, Rodionov DA, Ruckert C, Steiner J, Stevens R, Thiele I, Vassieva O, Ye Y, Zagnitko O, Vonstein V 2005 The subsystems approach to genome annotation and its use in the project to annotate 1000 genomes. Nucleic Acids Res 33:5691–5702. doi:10.1093/nar/gki866. 16214803 PMC1251668

[9] Strickman D, Sheer T, Salata K, Hershey J, Dasch G, Kelly D, Kuschner R 1995 *In vitro* effectiveness of azithromycin against doxycycline-resistant and -susceptible strains of *Rickettsia tsutsugamushi*, etiologic agent of scrub typhus. Antimicrob Agents Chemother 39:2406–2410. doi:10.1128/AAC.39.11.2406. 8585717 PMC162956

